# Protective effect of liquiritin on coronary heart disease through regulating the proliferation of human vascular smooth muscle cells via upregulation of sirtuin1

**DOI:** 10.1080/21655979.2021.2024687

**Published:** 2022-01-18

**Authors:** Liang Yuan, Dajie Wang, Chunyang Wu

**Affiliations:** a Department of Cardiology, First Affiliated Hospital of Nanjing Medical University, Nanjing, China; b Department of Cardiology, The Yancheng School of Clinical Medicine of Nanjing Medical University (Yancheng Third People’s Hospital), Yancheng, China

**Keywords:** Liquiritin, coronary heart disease, hVSMCs, SIRT1, proliferation, lactate dehydrogenase

## Abstract

This study aimed to explore whether liquiritin affects the development of coronary heart disease by regulating the proliferation and migration of human vascular smooth muscle cells (hVSMCs). A 3-(4,5-dimethylthiazol-2-yl)-2,5-diphenyl-2 H-tetrazolium bromide (MTT) assay and lactate dehydrogenase (LDH) release detection were performed to measure the toxic effects of liquiritin on hVSMCs. An *in vitro* atherosclerosis model in hVSMCs was established using oxidized low-density lipoprotein (ox-LDL), and cell proliferation and apoptosis were detected using an MTT assay and flow cytometry analysis. Western blotting and reverse transcriptase quantitative polymerase chain reaction (RT-qPCR) were used to detect protein and mRNA expressions, respectively. Caspase3 activity and cell migration were measured using an activity detection kit and Transwell assay, respectively. The results indicated that liquiritin at doses <160 μM had no significant effect on cell viability and LDH release in hVSMCs. Ox-LDL significantly induced cell proliferation and migration, and inhibited hVSMCs apoptosis. Liquiritin significantly inhibited cell proliferation and migration, and enhanced cell apoptosis in ox-LDL induced hVSMCs. Sirtuin1 (SIRT1) was lowly expressed in atherosclerotic plaque tissues in coronary heart disease patients and in ox-LDL-induced hVSMCs. Liquiritin improved SIRT1 expression in ox-LDL-induced hVSMCs, whereas the improvement was inhibited by Selisistat (EX 527, an effective SIRT1 inhibitor) treatment. EX 527 reversed the effects of liquiritin on cell proliferation, migration, and apoptosis in ox-LDL-induced hVSMCs In conclusion, liquiritin plays a protective role in coronary heart disease by regulating the proliferation and migration of hVSMCs by increasing SIRT1 expression.

## Introduction

Coronary atherosclerotic heart disease, also known as ischemic heart disease, refers to a type of heart disease caused by myocardial ischemia and hypoxia [1,2]. The coronary artery, the only blood vessel that supplies blood to the heart, hardens like the blood vessels of the whole body, exhibiting atherosclerotic changes, causing blood circulation disorders that feed the heart, as well as myocardial ischemia and hypoxia, which is a coronary heart disease [3,4]. Coronary heart disease is a common and frequently occurring disease in middle-aged and elderly people that severely endangers human life. Age, sex, high blood pressure, hyperlipidemia, obesity, and eating habits are common risk factors for coronary heart disease [5,6]. Atherosclerosis is a chronic inflammatory disease characterized by lipid accumulation, smooth muscle cell proliferation, apoptosis, necrosis, fibrosis, and local inflammation [7]. Immune and inflammatory responses have a significant impact on all stages of atherosclerosis. Increasing evidence shows that immunity plays a more important role in atherosclerosis by strictly regulating atherosclerosis progression [8–10]. The pathogenesis of atherosclerosis begins with functional changes in endothelial cells, and the most important cells are smooth muscle cells [11,12]. As one of the main parts of the arterial wall, vascular smooth muscle cells (VSMC) have many different structures and physical functions [13]. It has been reported that 70% of all cells in atherosclerotic lesions are derived from smooth muscle cells [14]. It has been reported that the accumulation of human vascular smooth muscle cells (hVSMCs) is related to the pathogenesis of atherosclerosis [15,16], therefore, we used hVSMCs in this study for a series of experiments.

Liquiritin, one of the primary flavonoids, is the main component of licorice and has various pharmacological activities [17,18]. Studies have found that liquiritin plays an important role in multiple diseases. Li et al. reported that liquiritin could reduce inflammation by inhibiting MAPK and TLR4/MyD88 signaling pathways, thereby reducing tissue damage [19]. A previous study reported that liquiritin exerted a neuroprotective effect on mouse brain injury induced by cerebral ischemia/reperfusion through antioxidant and anti-apoptotic mechanisms [20]. Study also revealed that liquiritin played a protective role in endothelial dysfunction induced by Advanced glycation end products (AGEs), and may be a promising drug for the treatment of vascular disease in diabetic patients [21]. However, there have been no reports on the effects of liquiritin on coronary heart disease.

SIRT1 is considered an anti-atherosclerotic factor [22,23]. SIRT1 activators is tested as the treatment of coronary artery disease [24]. Multiple studies have shown that SIRT1 can alleviate endothelial cell dysfunction induced by ox-LDL [25,26]. However, the role of SIRT1 in hVSMCs induced by ox-LDL is unclear.

In this study, we hypothesized that liquiritin might regulate the proliferation and migration of hVSMCs by regulating SIRT1 expression. Thus, this study was designed to explore whether liquiritin can regulate the proliferation and migration of hVSMCs by regulating SIRT1 expression to impact the development of coronary heart disease, which would provide new targets for coronary heart disease treatment.

## Materials and methods

### Cell culture and treatment

hVSMCs were obtained from American Type Culture Collection (Manassas, VA, USA). The cells were cultured in F-12 K medium (Gibco, TX, USA) containing 10% fetal bovine serum (FBS; Gibco) and incubated at 37°C and 5% CO_2_. The cells were treated with different liquiritin (ChengDu Conbon Bitech Co. LTD., Chengdu, China) concentrations (0, 1.25,2.5,5, 10, 20, 40, 80, and 160 μM) for 24 h, and then collected for further experiments.

### Tissue acquisition

Thirty cases of porridge-like sclerosis vascular plaque tissues and normal vascular tissues were collected from 30 coronary heart disease patients at Yancheng Third People’s Hospital. The study was approved by the medical ethics committee of Yancheng Third People’s Hospital, and written informed consent was obtained prior to tissue collection.

### MTT assay [27]

hVSMCs were plated in a 96-well plate (4x10^3^ cells/well) overnight, and 10 μL of MTT solution (Beyotime, Jiangsu, China) was added to each well and incubated with the cells for 4 h. After removing the medium, 100 μL dimethyl sulfoxide was added to each well to dissolve the formazan product, and the absorbance was recorded for 10 min at 570 nm using a microplate reader (Bio-Rad, CA, USA).

### Detection of LDH release [28]

The release of lactate dehydrogenase (LDH) was measured using an LDH Cytotoxicity Assay Kit (Beyotime). Briefly, cells were cultured in 96-well plates, and LDH release reagent was added into the wells 1 h prior to detection. We added 60 μL LDH detection working fluid to each well, and the cells were incubated in the dark for 30 min at room temperature. The absorbance was recorded at 490 nm using a microplate reader (Bio-Rad). LDH activity was presented as the fold of the control group.

### Establishment of atherosclerosis model in vitro

hVSMC cells were treated with 100 μg/mL ox-LDL for 24 h to establish an atherosclerosis model *in vitro* [29]. Cells without any treatment were used as the control.

To study the effect of liquiritin on ox-LDL induced hVSMCs, hVSMCs were treated with 10, 40, and 80 μM liquiritin [19] in the presence of 100 μg/mL ox-LDL for 24 h.

To explore whether SIRT1 was involved in the effect of liquiritin on ox-LDL induced hVSMCs, Selisistat (EX 527), an effective SIRT1 inhibitor was used [30]. hVSMCs were treated with 80 μM liquiritin or/and 10 μM EX 527 in the presence of 100 μg/mL ox-LDL for 24 h.

### Flow cytometry analysis [31]

Flow cytometry (FCM) analysis was performed to measure apoptosis in hVSMCs. Cells were resuspended in 1x buffer, and 195 μL Annexin V-FITC binding buffer was added after the supernatant was removed. Then, 5 μL Annexin V-FITC (Thermo Fisher Scientific, MA, USA) was added to the suspension and incubated at room temperature for 10 min in the dark. The supernatant was removed after incubation, and 190 μL Annexin V-FITC binding buffer was added to resuspend the cells, and then 10 μL propidium iodide staining solution (Thermo Fisher Scientific) was added. The stained cells were analyzed using a FACSCalibur flow cytometer (BD Biosciences, NJ, USA), and the data were analyzed using FlowJo software.

### Western blot analysis [32]

hVSMCs and tissues were lysed with radioimmunoprecipitation assay (RIPA) buffer (Beyotime) and centrifuged at 4°C to extract total protein. Protein concentration was detected using a bicinchoninic acid (BCA) kit (Bio-Rad). The protein was separated by sodium dodecyl sulfate-polyacrylamide gel electrophoresis (SDS-PAGE) and transferred to a polyvinylidene fluoride (PVDF) membrane. The membrane was incubated with Bcl-2 (cat. no. 4223; 1: 1000; Cell Signaling Technology, Inc.), Bax (cat. no. 5023; 1: 1000; Cell Signaling Technology, Inc.), MMP9 (cat. no. 13,667; 1: 1000; Cell Signaling Technology, Inc.), SIRT1 (cat. no. 2496; 1: 1000; Cell Signaling Technology, Inc.), or GAPDH (cat. no. 5174; 1: 1000; Cell Signaling Technology, Inc.) antibody at 4°C overnight after blocking with 5% nonfat milk phosphate buffer saline (PBS)-0.1%Tween 20 solution for 1 h. The membrane was then incubated with secondary antibodies (cat. no. ab96899; 1:2,000; Abcam, Cambridge, USA) for 1 h at room temperature. The protein bands were visualized using enhanced chemiluminescence luminescent substrate (Amersham ImageQuant800UV; Cytiva, MA, USA) and quantified using ImageJ software.

### Detection of caspase-3 activity [33]

hVSMCs were seeded into 6 well plates (5x10^4^ cells/well) and cultured at 37°C for 24 h. Then, the hVSMCs were treated with specific concentration of liquiritin or/and 10 μM EX 527 for 24 h. Subsequently, hVSMCs were collected by centrifugation at 600 g at 4°C for 5 min, and caspase-3 activity was determined using a colorimetric assay kit (Beyotime) according to the manufacturer’s specifications.

### Transwell assay [34]

A transwell assay was performed to detect cell migration ability using a Matrigel-free chamber. The cells were transferred to the upper chambers (8-μm pore size; Thermo Fisher Scientific) and cultured in serum-free medium after transfection for 48 h. Simultaneously, medium containing 10% FBS was added to the lower chambers. After 24 h, the migrated cells on the lower surface were fixed with 4% paraformaldehyde, stained with 0.1% crystal violet for 15 min, and imaged after removing non-migrated cells on the upper surface.

### RNA extraction and quantitative real-time PCR (RT-qPCR)

Total RNA from cells and tissues was extracted using TRIzol reagent (Invitrogen, MA, USA) and was reverse transcribed into cDNA using a cDNA synthesis kit (Thermo Fisher). The reaction conditions for reverse transcription were as following: 25°C for 5 min, 42°C for 60 min and 80°C for 2 min. cDNA was used for RT-qPCR analysis using the ChamQTM Universal SYBR® qPCR Master Mix (Vazyme, Nanjing, China). The amplification conditions were as follows: Pre-denaturation at 95°C for 10 min; followed by 38 cycles of denaturation at 95°C for 10 sec, annealing at 60°C for 20 sec and extension at 72°C for 34 sec. The primers were synthesized by Sangon Biotech Co., Ltd. (Shanghai, China) and listed as following: GAPDH, forward 5ʹ-CTTTGGTATCGTGGAAGGACTC-3ʹ and reverse 5ʹ-GTAGAGGCAGGGATGATGTTCT-3ʹ; MMP9,, forward 5ʹ-TGTACCGCTATGGTTACACTCG-3ʹ and reverse 5ʹ-GGCAGGGACAGTTGCTTCT-3ʹ; SIRT1,, forward 5ʹ-TAGCCTTGTCAGATAAGGAAGGA-3ʹ and reverse 5ʹ-ACAGCTTCACAGTCAACTTTGT-3ʹ.The relative mRNA expression levels of MMP9 and SIRT1 were calculated using the 2^−ΔΔCt^ analysis [35].

### Statistical analysis

Data are presented as mean ± standard deviation. The statistical analysis between each group was performed using Student’s t-test or one-way analysis of variance. Statistical significance was set at p < 0.05.

## Results

### Effects of liquiritin on cell viability in hVSMCs

**Figure 1.**
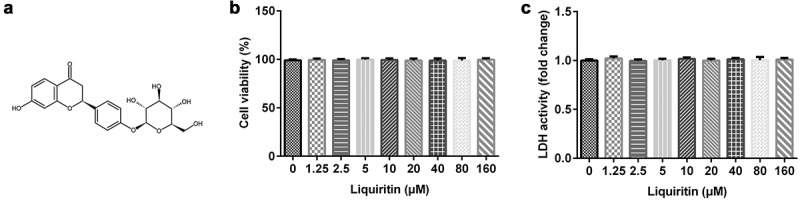
**Effects of liquiritin on cell viability in** human vascular smooth muscle cells **(hVSMCs)**. (a) The chemical formula of liquiritin. (b) The viability of hVSMCs was measured using an MTT assay. (c) The lactate dehydrogenase (LDH) release of hVSMCs was detected using an LDH Cytotoxicity Assay Kit.

Firstly, in order to study the toxic effect of liquiritin on hVSMCs, MTT and LDH assay were performed. Figure 1(a) shows the chemical formula of liquiritin, and the cell viability and LDH release were detected using an MTT assay and LDH Cytotoxicity Assay Kit after the cells were treated with different concentrations of liquiritin for 24 h. As shown in Figure 1(b,c), 0, 1.25, 2.5, 5, 10, 20, 40, 80, 160 μM liquiritin has no significant effect on the cell viability and LDH release of hVSMC cells. We selected 10, 40, and 80 μM liquiritin for the following experiments.

### Effects of liquiritin on cell proliferation in ox-LDL-induced hVSMCs

**Figure 2.**
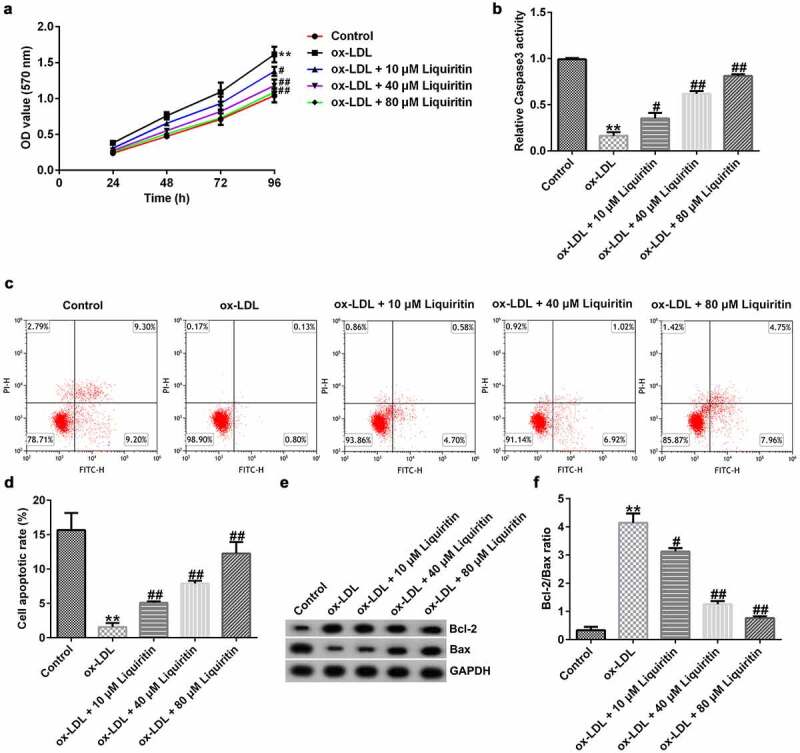
**Liquiritin reverses the proliferation of ox-LDL-induced hVSMCs**. (a) An MTT assay was performed to measure the cell proliferation of ox-LDL-induced hVSMCs. (b) Caspase-3 activity of ox-LDL-induced hVSMCs was determined using the colorimetric assay kit. (c, d) The cell apoptosis rates of ox-LDL-induced hVSMCs were measured by flow cytometry (FCM) analysis. (e, f) The protein expression levels of Bcl-2 and Bax in ox-LDL-induced hVSMCs were detected by Western blot analysis and quantified by ImageJ software. **P < 0.01 vs. Control; #^,^ ## P < 0.05, 0.01 vs. ox-LDL group.

To explore the effect of liquiritin on atherosclerosis, we established an atherosclerosis model *in vitro* using ox-LDL and detected the cell proliferation and apoptosis rate in hVSMCs by MTT and FCM analysis, respectively. The results showed that, compared with that of the control group, hVSMC proliferation in the ox-LDL group was significantly increased and the caspase-3 activity and hVSMC apoptosis in the ox-LDL group was significantly reduced, while liquiritin inhibited the proliferation and promoted the apoptosis of ox-LDL-induced hVSMCs in a dose-dependent manner (Figure 2(a–d)). Moreover, the protein expression levels of Bcl-2 and Bax were detected. As shown in Figure 2(e,f), in comparison with that of the control group, Bcl-2 protein expression and the Bcl-2/Bax ratio of hVSMCs in the ox-LDL group are remarkably increased, while Bax protein expression is significantly decreased. All these effects on hVSMCs were significantly reversed by liquiritin treatment.

### Effects of liquiritin on cell migration in ox-LDL-induced hVSMCs

**Figure 3.**
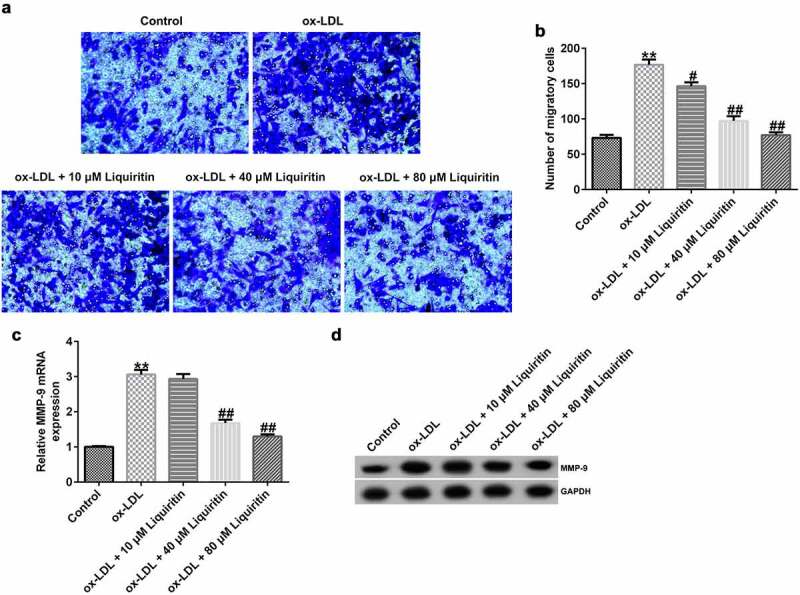
**Liquiritin reverses the migration of ox-LDL-induced hVSMCs**. (a, b) Transwell assay was performed to evaluate the migration ability of ox-LDL-induced hVSMCs. (c) MMP9 mRNA expression in ox-LDL-induced hVSMCs was detected by RT-qPCR analysis. (d) MMP9 protein expression in ox-LDL-induced hVSMCs was measured by Western blot analysis. **P < 0.01 vs. Control; #^,^ ## P < 0.05, 0.01 vs. ox-LDL group.

Transwell assay, Western blot analysis, and RT-qPCR were performed to study the effects of liquiritin on cell migration in hVSMCs. The results showed that, compared with that of the control group, the cell migration, MMP9 protein expression, and MMP9 mRNA expression in hVSMCs in the ox-LDL group were significantly increased, while liquiritin inhibited cell migration, and MMP9 mRNA expression, and MMP9 protein expression in ox-LDL-induced hVSMCs in a dose-dependent manner (Figure 3(a–d)).

### Effects of liquiritin on SIRT1 expression in ox-LDL-induced hVSMCs

**Figure 4.**
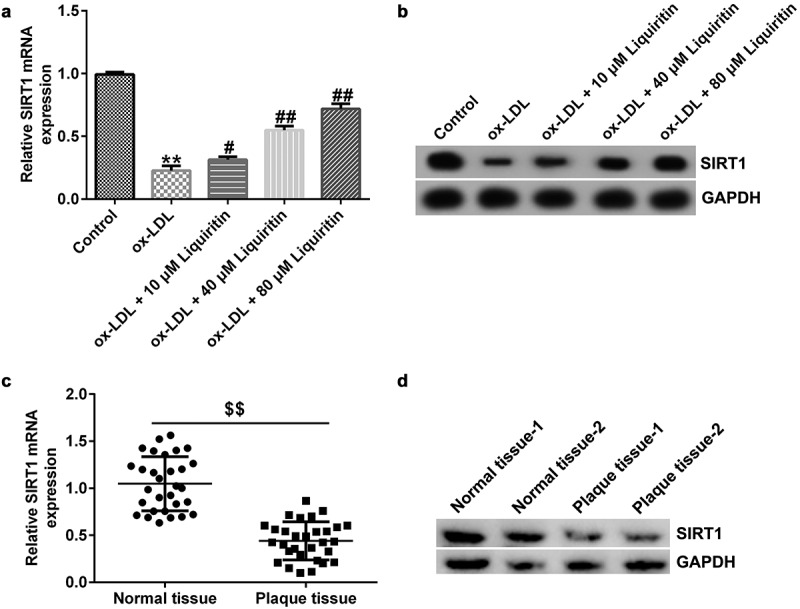
**Liquiritin increases SIRT1 expression in ox-LDL-induced hVSMCs**. The mRNA expression levels of SIRT1 in ox-LDL-induced hVSMCs were detected by RT-qPCR analysis. (b) The protein expression levels of SIRT1 in ox-LDL-induced hVSMCs were detected by Western blot analysis. (c) The mRNA expression levels of SIRT1 in plaque and normal tissues of 30 coronary heart disease patients were detected by RT-qPCR analysis. (d) The protein expression levels of SIRT1 in plaque and normal tissues of two random coronary heart disease patients were detected by Western blot analysis. **P < 0.01 vs. Control; #^,^ ## P < 0.05, 0.01 vs. ox-LDL group; $$ P < 0.01 vs. Normal tissue.

To study the effect of liquiritin on SIRT1 expression in coronary heart disease, we measured the mRNA and protein expression levels of SIRT1 in hVSMCs and patient-related tissues. Compared with that of the control group, SIRT1 expression in hVSMCs in the ox-LDL group was significantly reduced, while liquiritin increased SIRT1 expression in a dose-dependent manner (Figure 4(a,b)). Similarly, SIRT1 expression in atherosclerotic plaque tissues of coronary heart disease patients was significantly reduced compared to that in normal tissues (Figure 4(c,d)).

### Effects of SIRT1 on the growth of ox-LDL-induced hVSMCs

**Figure 5.**
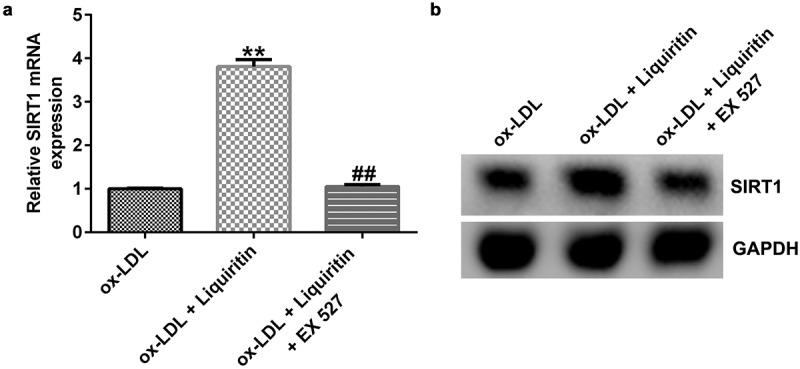
**EX 527 inhibits the increase of SIRT1 expression induced by liquiritin in ox-LDL-induced hVSMCs**. hVSMCs were treated with 80 μM liquiritin or/and 10 μM EX 527 in the presence of 100 μg/mL ox-LDL for 24 h. (a) The mRNA expression of SIRT1 in ox-LDL-induced hVSMCs was detected by RT-qPCR analysis. The fold change calculated against ox-LDL group. (b) The protein expression of SIRT1 in ox-LDL-induced hVSMCs was detected by Western blot analysis. **P < 0.01 vs. ox-LDL; ^##^ P < 0.05, 0.01 vs. ox-LDL+liquiritin group.

**Figure 6.**
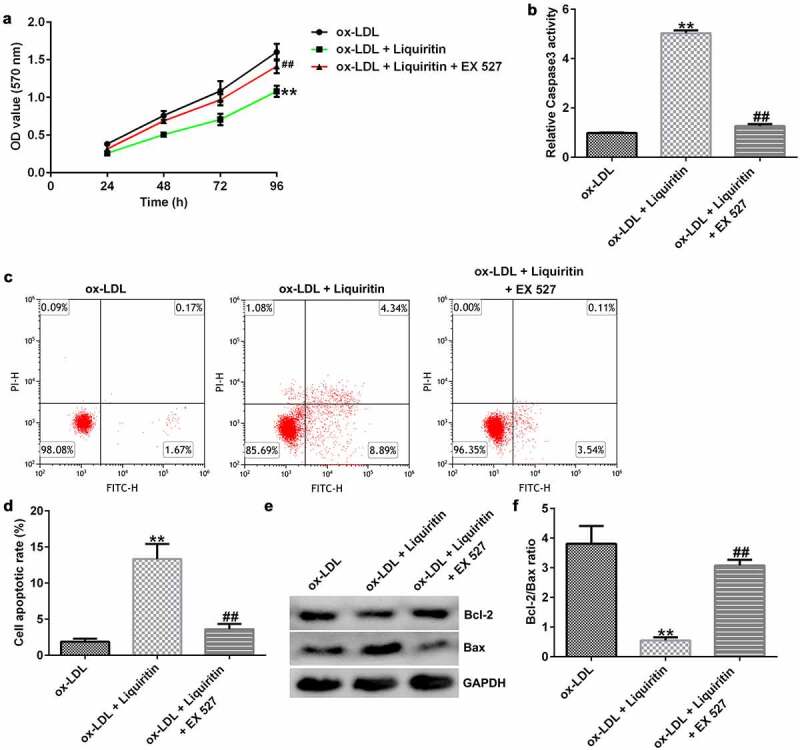
**EX 527 reverses the effects of liquiritin on cell proliferation in ox-LDL-induced hVSMCs**. hVSMCs were treated with 80 μM liquiritin or/and 10 μM EX 527 in the presence of 100 μg/mL ox-LDL for 24 h. An MTT assay was performed to measure the proliferation of ox-LDL-induced hVSMCs. (b) Caspase-3 activity of ox-LDL-induced hVSMCs was determined using a colorimetric assay kit. (c, d) The cell apoptosis rates of ox-LDL-induced hVSMCs were measured by FCM analysis. (e, f) The protein expression levels of Bcl-2 and Bax in ox-LDL-induced hVSMCs were detected by Western blot analysis and quantified using ImageJ software. **P < 0.01 vs. ox-LDL; ^##^ P < 0.05, 0.01 vs. ox-LDL+liquiritin group.

**Figure 7.**
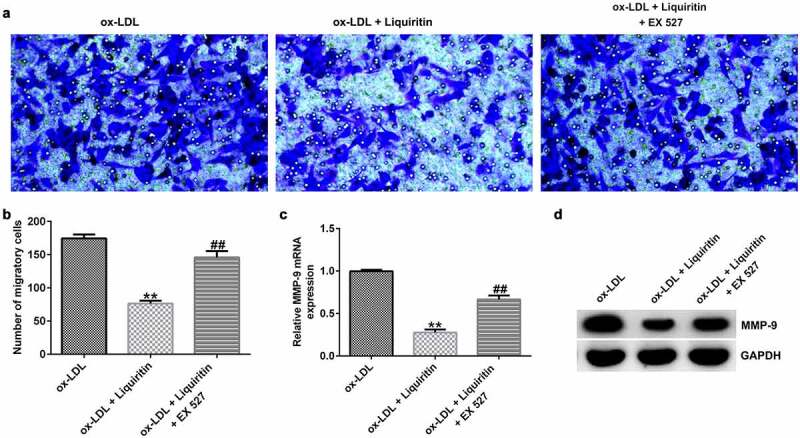
**EX 527 reverses the effects of liquiritin on cell migration in ox-LDL-induced hVSMCs**. hVSMCs were treated with 80 μM liquiritin or/and 10 μM EX 527 in the presence of 100 μg/mL ox-LDL for 24 h. (a, b) The migration ability of the treated hVSMCs was evaluated by Transwell assay. (c, d) MMP9 mRNA and protein expression in the treated ox-LDL-induced hVSMCs was measured by RT-qPCR and Western blot analysis. **P < 0.01 vs. ox-LDL; ## P < 0.05, 0.01 vs. ox-LDL+liquiritin group.

To investigate the effects of SIRT1 on the growth of ox-LDL-induced hVSMCs, the cells were treated with Selisistat (EX 527, SEN0014196), an effective SIRT1 inhibitor, and then collected for subsequent experiments. As shown in Figure 5(a,b), SIRT1 expression in hVSMCs of the ox-LDL+liquiritin group is significantly higher than that in the ox-LDL group, and this increase is significantly inhibited by EX 527. Compared to that of the ox-LDL group, the proliferation, Bcl-2 expression, and Bcl-2/Bax ratio of hVSMC cells in the ox-LDL+liquiritin group were significantly reduced, while caspase-3 activity, apoptosis, and Bax expression were markedly increased, and all the changes were reversed by EX 527 treatment (Figure 6(a–f)). Moreover, compared with that of the ox-LDL group, the cell migration and the expression of MMP9 mRNA and protein of hVSMCs in ox-LDL+liquiritin group were significantly reduced, and these changes could be reversed by EX 527 treatment (Figure 7(a–d)).

## Discussion

Coronary heart disease refers to the deposition of lipids and other blood components in the arterial intima, smooth muscle cell proliferation, and an increase in collagen fibers, forming atherosclerotic fat-containing necrotic lesions and hardening of the vascular wall [6,36]. The majority of patients have no symptoms in the early stage, and they may already have more serious atherosclerosis when they have more obvious symptoms. Therefore, there is an urgent need to identify an effective coronary heart disease treatment. It has been reported that cell proliferation and migration of hVSMCs are closely related to the occurrence of coronary heart disease [37], and the abnormal proliferation and migration of vascular smooth muscle cells is one of the pathogenesis of atherosclerosis [15,16]. Liquiritin is considered to have various pharmacological activitiess [17,18,38]. And liquiritin has been reported to be a promising agent for the treatment of vascular disease in diabetic patients [21]. In this study, we suspected that liquiritin would participate in the progression of coronary heart disease.

First, we investigated the side effects of liquiritin on hVSMCs and found that liquiritin at doses <160 μM had no significant effect on cell viability and LDH release in hVSMCs. We established an atherosclerosis model *in vitro* in hVSMCs induced by 100 μg/mL ox-LDL [29], and measured the effects of liquiritin at different concentrations on cell proliferation, apoptosis, and migration. MMP-9, a member of the endopeptidase family, is involved in the degradation and remodeling of the extracellular matrix [39]. During vascular injury and inflammation, the increased expression of MMP-9 in vascular component cells plays a key role in the formation and rupture of atherosclerotic plaques [40]. In addition, MMP-9 also plays an important role in VSMC proliferation and migration [41]. Thus, MMP-9 expression was analyzed in current study. Consistent with previous results [29], our study shows that ox-LDL promotes the proliferation and migration of hVSMCs. As expected, the results showed that liquiritin inhibited the proliferation, enhanced apoptosis, suppressed migration, as well as promoted MMP-9 expression in ox-LDL-induced hVSMCs in a dose-dependent manner.

We performed further experiments to further study the mechanism of action of liquiritin in the development of coronary heart disease. Recent studies have shown that SIRT1 plays a complex physiological role in regulating glucose and lipid metabolism, inflammation, oxidative stress, cell aging, and apoptosis [42,43]. It also plays an important regulatory role in the occurrence and development of atherosclerosis [44,45]. Therefore, we speculated that liquiritin may play a role in ox-LDL-induced hVSMCs through regulating SIRT1 expression. We verified this conjecture through experiments. The results showed that SIRT1 was weakly expressed in the atherosclerosis model *in vitro*, and liquiritin increased SIRT1 expression in ox-LDL-induced hVSMCs. Moreover, all the effects of liquiritin on ox-LDL-induced hVSMCs were at least partly reversed by SIRT1 inhibition.

Overall, liquiritin inhibited the effects of ox-LDL on hVSMCs by increasing SIRT1 expression, thus may play a protective role in coronary heart disease. However, this study was only a preliminary *in vitro* study of the effect of liquiritin on coronary heart disease. In order to make the influence of liquiritin on coronary heart disease more convincing, effects of liquiritin on coronary heart disease should be investigated in animal models. We will perform this issue in our next research.

## Conclusion

Liquiritin plays a protective role in coronary heart disease by regulating the proliferation and migration of hVSMCs by increasing SIRT1 expression, which would provide new ideas for coronary heart disease treatment.

## Supplementary Material

Supplemental Material

## Data Availability

All datasets used and/or generated during the current study are available from the corresponding author upon reasonable request.

## Research highlights

- Liquiritin inhibited cell proliferation in ox-LDL-induced hVSMCs.
- Liquiritin suppressed cell migration in ox-LDL-induced hVSMCs.
- Liquiritin affected ox-LDL-induced hVSMCs via up-regulating SIRT1 expression.
