# Supplementary material for: Protective effect of liquiritin on coronary heart disease through regulating the proliferation of human vascular smooth muscle cells via upregulation of sirtuin1
Source: Bioengineered. 2022 Jan 18;13(2):2840–50. doi: 10.1080/21655979.2021.2024687 (PMC8974169; doi:10.1080/21655979.2021.2024687)
Supplement: Supplemental Material [file KBIE_A_2024687_SM7361.zip › supplementary/Supplemental file2.zip!/original blots of Western blot/Special Note.docx]

Dear editor,

What we need to explain is: in the process of Western blot assay, we first cut out the corresponding membrane according to the molecular weight of the target protein, and then incubate the antibody. So our original strip is cut. But we guarantee that all our results are obtained through scientific and rigorous experiments. We strictly follow the Western blot assay procedure for experiments. Hope to get your understanding and acceptance.

Best regards
